# A nomogram-based clinical prediction model for adverse clinical outcomes in non-HIV Pneumocystis *jirovecii* pneumonia patients

**DOI:** 10.1186/s12890-025-03700-2

**Published:** 2025-05-17

**Authors:** Dong Wang, Lujia Guan, Qian Yin, Xiaoxia Hou, Xi Zhan, Zhaohui Tong

**Affiliations:** 1 Department of Respiratory and Critical Care Medicine, Beijing Research Center for Respiratory Infectious Diseases, Beijing Institute of Respiratory Medicine and Beijing Chao-Yang Hospital, Capital Medical University, Beijing, China; 2 Department of Neurology, Beijing Chao-Yang Hospital, Capital Medical University, Beijing, China; 3 Department of General Medicine, Beijing Chao-Yang Hospital, Capital Medical University, Beijing, China

**Keywords:** Pneumocystis *jirovecii* pneumonia, Predictive nomogram, Adverse clinical outcomes

## Abstract

**Background:**

Non-human immunodeficiency virus (HIV) immunocompromised patients with Pneumocystis *jirovecii* pneumonia (PJP) face rapid progression and high mortality, necessitating a predictive model to identify patients at risk of adverse clinical outcomes for timely interventions and improved stratification.

**Methods:**

Patients admitted between January 2011 and June 2024 at Beijing Chao-Yang Hospital were retrospectively analyzed. Collected data included patients’ demographics, smoking status, comorbidities, immunosuppressive diseases, blood laboratory tests, in-hospital treatment, and adverse clinical outcomes. Predictor selection was performed using the least absolute shrinkage and selection operator (LASSO) and logistic regression, with selected features incorporated into a nomogram. Internal validation was conducted using a 500-bootstrap resampling method to ensure model robustness. Model performance was assessed via the area under the receiver operating curve (AUC), calibration plots, decision curve analysis (DCA), and clinical impact curve (CIC).

**Results:**

Among the 431 patients, 243 (56.4%) experienced adverse clinical outcomes. LASSO regression screened 21 variables, selecting 9 predictors with non-zero coefficients through 10-fold cross-validation at lambda.1se = 0.0453 (log(lambda.1se) = -3.092). Multivariate logistic regression identified 7 independent risk factors for adverse clinical outcomes: smoking status, cytomegalovirus infection, diabetes, neutrophil-lymphocyte ratio (NLR), lactate dehydrogenase (LDH), PaO_2_/FiO_2_ (PFR), and lymphocyte subset. These factors were incorporated into a nomogram, achieving an AUC of 0.89 (95% CI: 0.86–0.92), with the Hosmer–Lemeshow test (*p* = 0.134) and calibration curves showing strong agreement between predicted and observed outcomes. Internal validation via 500-bootstrap resampling yielded a bias-corrected AUC of 0.83 (95% CI: 0.80–0.86). DCA demonstrated strong clinical decision-making utility, while the CIC confirmed its practical reliability.

**Conclusions:**

Regression analysis identified smoking status, CMV infection, diabetes, NLR, LDH, PFR, and lymphocyte subset as independent risk factors for adverse clinical outcomes in non-HIV PJP patients. The predictive model constructed from these factors exhibited robust accuracy and reliability.

**Supplementary Information:**

The online version contains supplementary material available at 10.1186/s12890-025-03700-2.

## Introduction

Pneumocystis *jirovecii* pneumonia (PJP) is a life-threatening opportunistic fungus infection caused by a dysregulated host response and impaired microbial clearance due to immunosuppression [1, 2]. An epidemic phase of PJP was noted in the 1980s in HIV-infected patients, but its incidence significantly decreased following the development of highly active antiretroviral therapy (HAART) and trimethoprim-sulfamethoxazole (TMP-SMX) prophylaxis, with a steady decline in prevalence among hospital HIV-positive patients in the US. However, the decrease in infections among people with HIV has been offset by a rising incidence in newly at-risk groups, such as solid organ transplant recipients and individuals receiving advanced immunosuppressive therapies [3].

Unlike HIV-associated PJP, clinical manifestations in non-HIV patients are non-specific (fever, cough, and dyspnea), with rapid disease progression to respiratory failure [4]. More than half of non-HIV PJP patients require admission to the intensive care unit (ICU), with mortality rates ranging from 50–60% [5]. Various prognostic indicators, including solid malignancies, lymphocytes, lactate dehydrogenase (LDH), C-reactive protein (CRP), platelet, and neutrophil-to-lymphocyte ratio (NLR) have been associated with poor clinical outcomes in non-HIV PJP patients [6–8]. Non-HIV PJP is further characterized by significant heterogeneity in immunosuppression severity, treatment regimens, and disease progression, which complicates early risk stratification and timely intervention.

Given these challenges, accurate predictive tools are essential to enhance clinical decision-making and improve outcomes in this vulnerable population. In this study, we developed and validated a predictive model to assess the risk of adverse clinical outcomes, including ICU admission, invasive mechanical ventilation (IMV), in-hospital mortality, and mortality within 28 days, in non-HIV PJP patients. By integrating clinical and laboratory parameters, this model aims to support early risk stratification, guide timely interventions, and optimize patient management from the start of hospitalization.

## Materials and methods

### Study population

A retrospective cohort study was conducted in China at Beijing Chao-Yang Hospital from January 2011 to June 2024 to derive and validate a prediction model for non-HIV patients with PJP. HIV-negative participants were eligible for inclusion if they had a history of immunosuppression due to underlying conditions such as malignancies (including hematological malignancies and solid tumors), solid organ transplantation, connective tissue diseases, or other immunocompromised status, and had a diagnosis of proven or probable PJP. Patients with immunodeficiencies were not included in our study cohort. Exclusion criteria comprised: (1) patients aged under 18 years, (2) patients with P. *jirovecii* colonization, (3) patients diagnosed with COVID-19, (4) cases with missing clinical data, and (5) patients who had received intensive care in external hospitals’ ICUs. For patients with multiple admissions during the study period, only the initial admission record was analyzed. The study adhered to the principles of the Declaration of Helsinki and was approved by the Research Ethics Board of Beijing Chao-Yang Hospital (2021-ke-192). Due to its retrospective design, the requirement for written informed consent was waived.

### Data collection and outcome

Data were collected on demographic characteristics (age, sex), smoking status (never, former, and current smoke), comorbidities (diabetes, hypertension, chronic pulmonary disease), symptoms (dyspnea, sputum, cough, fever), along with immunosuppressive diseases (e.g., cancer, solid organ transplantation, hematological diseases), and vital signs (heart rate, systolic blood pressure, and diastolic blood pressure) at admission. Laboratory findings, including white blood cell (WBC) count, red blood cell count, CRP, hemoglobin levels, blood gas analysis (pH, PaO_2_, and PaCO_2_), and lymphocyte subsets, were recorded at the initial assessment. NLR and platelet-to-lymphocyte ratio (PLR) were calculated as the absolute neutrophil or platelet count divided by the absolute lymphocyte count, respectively. Additional respiratory pathogens detected from the same sample used for PJP diagnosis were collected to identify potential respiratory coinfections [9]. Pharmacotherapies during hospitalization, including adjunctive corticosteroids and TMP-SMX were used as covariates. Adverse clinical outcomes were defined as a composite of IMV, ICU admission, in-hospital mortality, and 28-day mortality.

### Definition

The diagnosis of PJP relies on a combination of clinical symptoms, including fever, cough, dyspnea, and hypoxemia, alongside radiological evidence of interstitial syndrome on a CT scan and detection of P. *jirovecii* in respiratory samples, such as induced sputum, bronchial aspirates, or bronchoalveolar lavage fluid (BALF). A definitive PJP diagnosis is made when P. *jirovecii* is identified through direct microscopic examination. Probable PJP is diagnosed when quantitative polymerase chain reaction (qPCR) detects P. *jirovecii* above laboratory-defined thresholds, supported by consistent clinical and radiological findings, despite negative microscopic examination. Colonization was defined by a positive qPCR result without fulfilling the clinical and/or radiological criteria for a proven or probable PJP diagnosis [7]. Delayed initiation of TMP-SMX treatment was defined as administration starting ≥ 96 h after admission [10].

### Statistical analysis

Categorical variables were summarized as frequencies and percentages, with group comparisons conducted using the χ² test or Fisher’s exact test, as appropriate. Continuous variables were expressed as medians with interquartile ranges (IQR) and analyzed using the Mann-Whitney U test. No imputation was applied in this study.

Univariate logistic regression was performed to evaluate the relationship between potential predictors and adverse clinical outcomes. Significant variables identified in univariate analysis were included in the subsequent least absolute shrinkage and selection operator (LASSO) regression to address multicollinearity and overfitting [11]. LASSO regression was implemented using the “glmnet” package in R, with 10-fold cross-validation to determine the optimal lambda value. The “lambda.1se” criterion was selected to achieve a balance between model performance and simplicity. The final predictive model was constructed using multivariable logistic regression, excluding variables with two-sided *P*-values > 0.05. The model’s discriminatory performance was assessed using AUC, and a visual nomogram was developed with the “VPRM” package in R to estimate the probability of adverse clinical outcomes based on predictor-specific scores [12]. Model calibration was evaluated using the Hosmer‒Lemeshow test and calibration curves, where a *p*-value > 0.05 indicated a good fit. Clinical utility was assessed through decision curve analysis (DCA) and clinical impact curve (CIC) analysis, which incorporated potential decision-making outcomes. Internal validation was performed using the bootstrap method with 500 resampled datasets.

All statistical analyses were conducted using R software version 4.4.1, with statistical significance defined as a two-sided *P*-value < 0.05.

## Results

### Characteristics of the study population

**Fig. 1:**
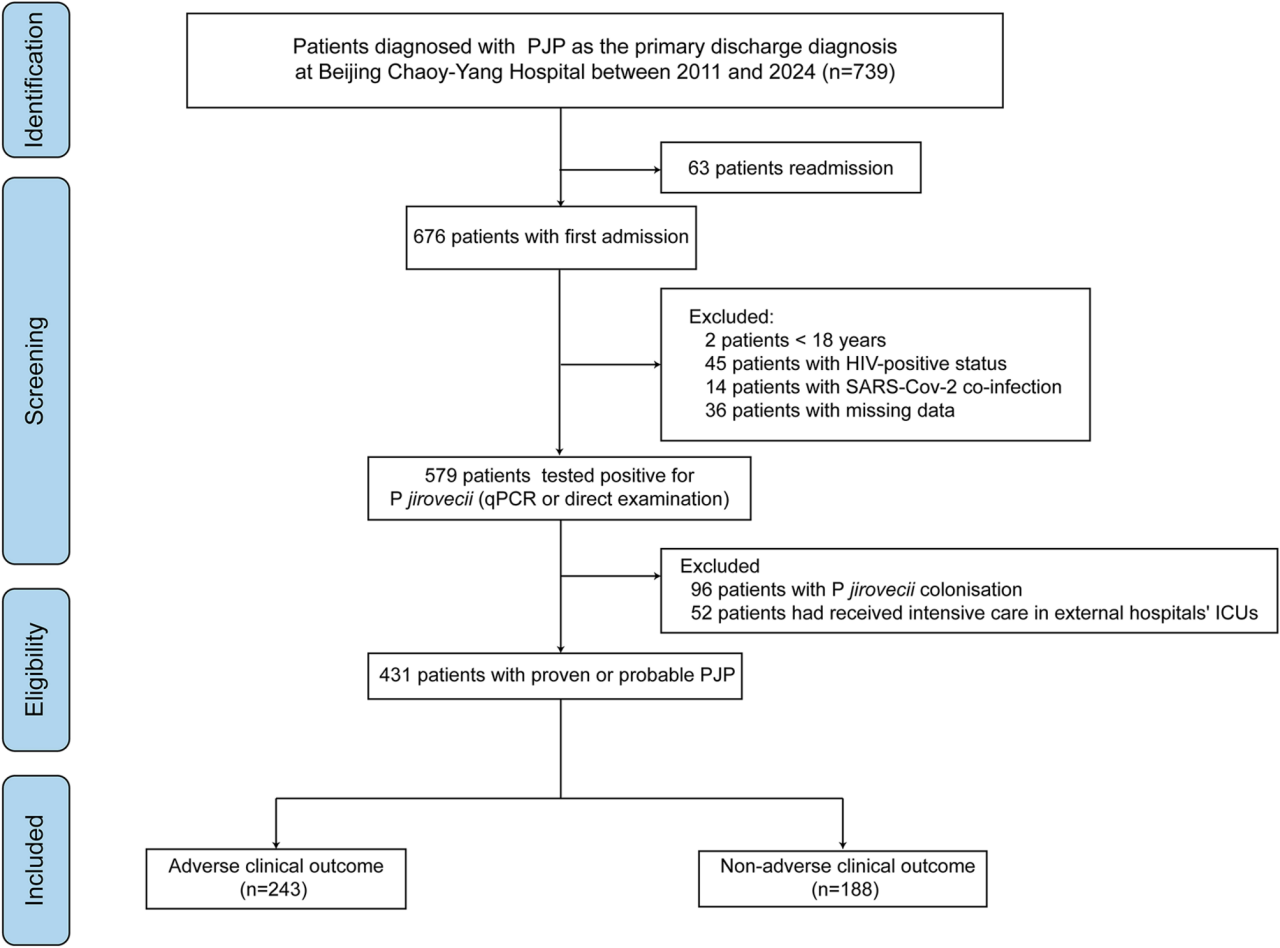
The flow chart of the study population Note: PJP, pneumocystis *jirovecii* pneumonia; HIV, human immunodeficiency virus; qPCR, quantitative polymerase chain reaction; ICU, intensive care unit

A total of 431 patients were included in this study, with 188 patients in the non-adverse clinical outcome group and 243 patients in the adverse clinical outcome group (Fig. 1). The classification of adverse clinical outcomes is detailed in Table 1 and Figure S1. The most frequently identified immunosuppressive conditions among the study population included connective tissue diseases (28.7%), followed by solid organ transplantation (17.0%), interstitial lung diseases (13.3%), and solid tumors (12.2%). Comparison of baseline data showed significant differences between the two groups in terms of age, smoking status, diabetes, dyspnea, fever, solid organ transplantation, hematological disease, heart rate, WBC, NLR, PLR, CRP, LDH, blood urea nitrogen (BUN), pH, PaO_2_/FiO_2_ ratio (PFR), CD4^+^ T cell, adjunctive corticosteroid, TMP-SMX, bacteria, cytomegalovirus (CMV), fungus (*P* < 0.05), as shown in Table 1.

**Table 1:** Comparison of clinical characteristics between patients with and without adverse clinical outcomes

Clinical characteristic	Non-adverse clinical outcomes (*n* = 188)	Adverse clinical outcomes (*n* = 243)	*P*-value
Age, years, median (IQR)	56 (45, 63)	62 (49, 71)	< 0.001
Sex, n (%)			0.144
Female	82 (43.6%)	88 (36.2%)	
Male	106 (56.4%)	155 (63.8%)	
Smoking status, n (%)			0.008
Never or former smoke	166 (88.3%)	190 (78.2%)	
Current smoke	22 (11.7%)	53 (21.8%)	
Comorbidities, n (%)			
Diabetes	42 (22.3%)	83 (34.2%)	0.010
Hypertension	82 (43.6%)	110 (45.3%)	0.807
Chronic pulmonary disease			0.862
COPD	7 (3.7%)	9 (3.7%)	
Asthma	4 (2.1%)	6 (2.5%)	
Bronchiectasis	5 (2.7%)	8 (3.3%)	
Symptoms, n (%)			
Dyspnea	82 (43.6%)	134 (55.1%)	0.022
Sputum	60 (31.9%)	91 (37.4%)	0.274
Cough	82 (43.6%)	99 (40.7%)	0.612
Fever	85 (45.2%)	139 (57.2%)	0.017
Immunosuppressive status, n (%)			
Solid organ transplantation	32 (17.0%)	73 (30.0%)	0.022
Cancer	23 (12.2%)	17 (7.0%)	0.091
Hematological disease	16 (8.5%)	39 (16.0%)	0.029
Interstitial lung disease	25 (13.3%)	20 (8.2%)	0.121
Connective tissue disease	54 (28.7%)	65 (26.7%)	0.729
Nephrotic syndrome	12 (6.4%)	16 (6.6%)	0.701
Other comorbidities	13 (6.9%)	10 (4.1%)	0.910
Pre-onset corticosteroid exposure.	135 (71.8%)	191 (78.6%)	0.129
Vital signs, median (IQR)			
Heart rate, rate/minute	84 (78, 98)	92 (80, 104)	< 0.001
Systolic blood pressure, mmHg	122 (112, 132)	123 (112, 135)	0.593
Diastolic blood pressure, mmHg	76 (68, 82)	75 (68, 80)	0.492
Laboratory results, median (IQR)			
WBC, 10^9^/L	6.5 (4.3, 9.3)	7.2 (5.0, 10.1)	0.031
NLR	6.7 (3.6, 11.1)	10.5 (7.2, 18.4)	< 0.001
Platelet, 10^9^/L	195 (151, 263)	192 (142, 245)	0.130
PLR	254 (172, 365)	321 (225, 479)	< 0.001
Erythrocytes, 10^12^/L	3.7 (3.1, 4.1)	3.6 (3.1, 4.2)	0.549
Hemoglobin, g/L	113 (95, 127)	110 (93, 127)	0.294
C-reactive protein, mg/dL	5.4 (1.8, 10.7)	8.5 (5.0, 13.5)	< 0.001
LDH, U/L	448 (375, 562)	524 (441, 656)	< 0.001
Creatinine, umol/L	81.4 (69.4, 101.1)	79.4 (70.1, 100.3)	0.855
BUN, mmol/L	7.2 (4.8, 9.8)	7.8 (5.9, 12.7)	0.008
Uric acid, umol/L	256.5 (192.4, 323.8)	258.0 (182.7, 357.0)	0.898
Prothrombin time, s	12.0 (10.4, 13.2)	11.5 (10.0, 12.8)	0.164
Fibrinogen, mg/dl	413.5 (333.5, 498.5)	426.1 (333.9, 532.9)	0.180
Blood gas analysis			
pH	7.45 (7.42, 7.47)	7.44 (7.41, 7.46)	0.045
PaCO_2_, mmHg	33.0 (28.9, 37.4)	34.0 (29.6, 40.0)	0.174
PFR, mmHg	384.0 (340.0, 416.0)	334.0 (311.2, 344.0)	< 0.001
CD4^+^ T cell, cell/ul	170 (128, 280)	94 (47, 154)	< 0.001
Treatment, n (%)			
TMP-SMX	34 (18.1%)	67 (27.6%)	0.006
Adjunctive corticosteroid	54 (28.7%)	96 (39.5%)	< 0.001
Co-infection, n (%)			
Bacteria	47 (25.0%)	86 (35.4%)	0.040
CMV	65 (34.6%)	126 (51.9%)	0.004
Fungus	38 (20.2%)	73 (30.0%)	0.027

Note: IQR Interquartile range; CMV, cytomegalovirus; WBC, white blood cell count; NLR, neutrophil–lymphocyte ratio; PLR, platelet-lymphocyte ratio; LDH, lactate dehydrogenase; BUN, blood urea nitrogen; PFR, PaO_2_/FiO_2_ ratio; TMP-SMX, trimethoprim-sulfamethoxazole

### LASSO analysis screening for potential predictors

In the univariate logistic regression analysis, several variables were found to be significantly associated with adverse clinical outcomes. These included demographic and comorbidity factors such as older age, current smoking, elevated heart rate, diabetes mellitus, solid organ transplantation, and hematological malignancies. Inflammatory markers, including elevated NLR, PLR, and CRP, as well as higher levels of LDH and BUN were also associated with worse outcomes. Additionally, clinical features such as dyspnea, fever, reduced PFR, lower CD4^+^ T cell counts, the use of adjunctive corticosteroids and TMP-SMX, and co-infections (bacterial, fungal, or cytomegalovirus) were significantly related to poor prognosis (Table 2). These variables were subsequently included in the LASSO regression model for further variable selection and dimensionality reduction prior to multivariate logistic regression.

**Table 2:** The univariate analyses of predicting factors for adverse clinical outcomes among patients hospitalized for Pneumocystis pneumonia

Variables	OR(95%CI)	*P* value	Variables	OR(95%CI)	*P* value
Age, years	1.028(1.015–1.043)	< 0.001	Nephrotic syndrome		
Sex			No	Reference	
Female	Reference		Yes	1.67(0.33–1.34)	0.256
Male	1.36(0.92–2.01)	0.119	Other comorbidities		
Smoke status			No	Reference	
Never or former smoke	Reference		Yes	0.60(0.28–1.29)	0.197
Current smoke	2.10(1.24–3.66)	< 0.001	Vital signs		
Comorbidities			Heart rate	1.013(1.004–1.024)	0.007
Diabetes mellitus			Systolic blood pressure	1.004(0.993–1.015)	0.451
No	Reference		Diastolic blood pressure	0.993(0.976–1.010)	0.475
Yes	1.80(1.17–2.79)	0.007	Laboratory results		
Hypertension			WBC	1.023(0.997–1.062)	0.138
No	Reference		NLR	1.073(1.046–1.104)	< 0.001
Yes	1.06(01.72–1.56)	0.733	Platelet	0.998(0.995–1.001)	0.074
Chronic pulmonary disease			PLR	1.001(1.000-1.002)	0.003
No	Reference		Erythrocytes	1.011(0.993–1.020)	0.631
Yes	1.12(0.57–2.22)	0.720	Hemoglobin	0.996(0.988–1.004)	0.421
Symptoms			C-reactive protein	1.038(1.014–1.066)	0.002
Dyspnea			LDH	1.004(1.003–1.006)	< 0.001
No	Reference		Creatinine	0.997(0.990–1.005)	0.498
Yes	1.69(1.15–2.50)	0.006	BUN	1.039(1.005–1.078)	0.038
Sputum			Uric acid	0.999(0.998–1.001)	0.909
No	Reference		Prothrombin time	0.985(0.947–1.025)	0.468
Yes	1.27(0.86–1.91)	0.232	Fibrinogen	1.000(0.999–1.002)	0.164
Cough			Blood gas analysis		
No	Reference		pH	0.592(0.224–1.198)	0.199
Yes	0.88(0.60–1.30)	0.548	PaCO_2_	1.017(0.999–1.037)	0.067
Fever			PFR	0.989(0.986–0.993)	< 0.001
No	Reference		CD4^+^ T cell	0.983(0.979–0.986)	< 0.001
Yes	1.61(1.10–2.38)	0.014	Treatment		
Immunosuppressive diseases			TMP-SMX		0.021
Solid organ transplantation			No	Reference	
No	Reference		Yes	1.72(1.09–2.77)	
Yes	2.09(1.31–3.37)	0.002	Adjunctive corticosteroid		0.020
Cancer			No	Reference	
No	Reference		Yes	1.62(1.08–2.44)	
Yes	0.53(0.27–1.03)	0.062	Co-infection		
Hematological disease			Bacteria		0.021
No	Reference		No	Reference	
Yes	2.05(1.12–3.90)	0.022	Yes	1.64(1.08–2.51)	
Interstitial lung disease			CMV		< 0.001
No	Reference		No	Reference	
Yes	0.91(0.59–1.38)	0.649	Yes	2.04(1.38–3.03)	
Connective tissue disease			Fungus		0.021
No	Reference		No	Reference	
Yes	0.82(0.53–1.2)	0.350	Yes	1.69(1.08–2.67)	
Pre-onset corticosteroid exposure					
No	Reference				
Yes	1.44(0.93–2.24)	0.104			

Note: OR, odd ratio; CI, confidential interval; WBC, white blood cell count; NLR, neutrophil–lymphocyte ratio; PLR, platelet-lymphocyte ratio; LDH, lactate dehydrogenase; BUN, blood urea nitrogen; PFR, PaO2/FiO2 ratio; TMP-SMX, trimethoprim-sulfamethoxazole; CMV, cytomegalovirus

**Fig. 2:**
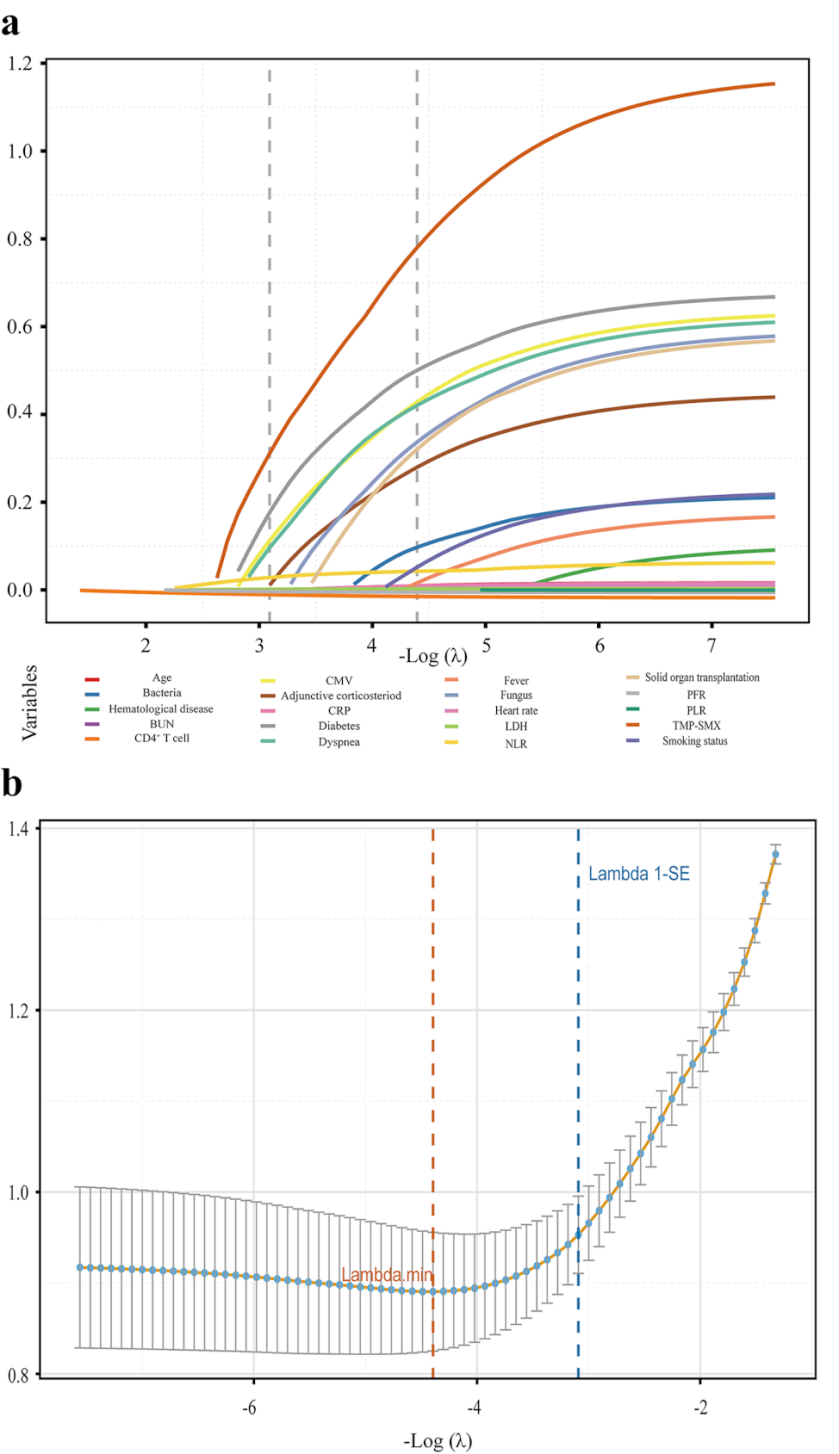
Potential predictors’ selection using the LASSO regression. (**a**) The LASSO regression analysis generated coefficient profiles for 20 candidate variables, visualized as a plot against the sequence of log(λ). (**b**) The optimal λ value was determined through 10-fold cross-validation, with dashed lines marking lambda.min and lambda.1se. The dotted orange line represents the λ value corresponding to the minimum deviance (lambda.min), while the blue dotted line corresponds to the 1 standard error criteria (lambda.1se) Abbreviations: BUN, blood urea nitrogen; CMV, cytomegalovirus; CRP, C-reactive protein; LDH, lactate dehydrogenase; NLR, neutrophil–lymphocyte ratio; PFR, PaO_2_/FiO_2_ ratio; PLR, platelet-lymphocyte ratio; TMP-SMX, trimethoprim-sulfamethoxazole

In the LASSO algorithm, changes in the lambda value were tracked to observe the behavior of predictor coefficients, reflecting their importance (Fig. 2a). At the minimum lambda value (lambda.min = 0.0123; log(lambda.min) =-4.3948), 17 variables with non-zero coefficients were identified. To achieve a balance between model simplicity and generalizability, appropriate lambda value (lambda.1se = 0.0453; log(lambda.1se) = -3.0923) was selected, yielding a more concise model with 9 key variables (Fig. 2b). These variables included smoking status, dyspnea, diabetes, NLR, LDH, PFR, CD4^+^ T cell, adjunctive corticosteroid use, and CMV infection.

### Multivariate logistic regression analysis

The outcome variable, adverse clinical outcomes, was analyzed using multivariate logistic regression incorporating the 9 selected characteristics as independent variables. The results showed that current smoking (OR: 2.870, 95% CI: 1.262–6.521, *P* = 0.012), CMV infection (OR: 1.731, 95% CI: 1.007–2.973, *P* = 0.047), and diabetes (OR: 2.198, 95% CI: 1.160–4.164, *P* = 0.016) were independent predictors. Key laboratory predictors included the NLR (OR: 1.064, 95% CI: 1.027–1.103, *P* < 0.001), LDH levels (OR: 1.003, 95% CI: 1.001–1.005, *P* < 0.001), PFR (OR: 0.994, 95% CI: 0.989–0.997, *P* = 0.002), and CD4^+^ T cell counts (OR: 0.981, 95% CI: 0.976–0.985, *P* = 0.005) were independent risk factors (Table 3).

**Table 3:** Multivariate logistic regression for adverse clinical outcome prediction in hospitalized patients with Pneumocystis pneumonia

Variables	Multivariate logistic regression	
	Adjusted OR (95% CI)	*P* value
Adjunctive corticosteroid		
No	Reference	
Yes	1.615 (0.900-2.899)	0.109
Smoking status		
Never or former smoke	Reference	
Current smoke	2.870 (1.262–6.521)	0.012
CMV		
No	Reference	
Yes	1.731 (1.007–2.973)	0.047
Dyspnea		
No	Reference	
Yes	1.689 (0.974–2.960)	0.062
Diabetes mellitus		
No	Reference	
Yes	2.198 (1.160–4.164)	0.016
NLR	1.064 (1.027–1.103)	< 0.001
LDH	1.003 (1.001–1.005)	< 0.001
PFR	0.994 (0.989–0.997)	0.002
CD4^+^ T cell	0.981 (0.976–0.985)	0.005

Note: OR, odd ratio; CI, confidential interval; CMV, cytomegalovirus; NLR, neutrophil–lymphocyte ratio; LDH, lactate dehydrogenase; PFR, PaO_2_/FiO_2_ ratio

### Creation and evaluation of adverse clinical outcomes prediction nomogram

**Fig. 3:**
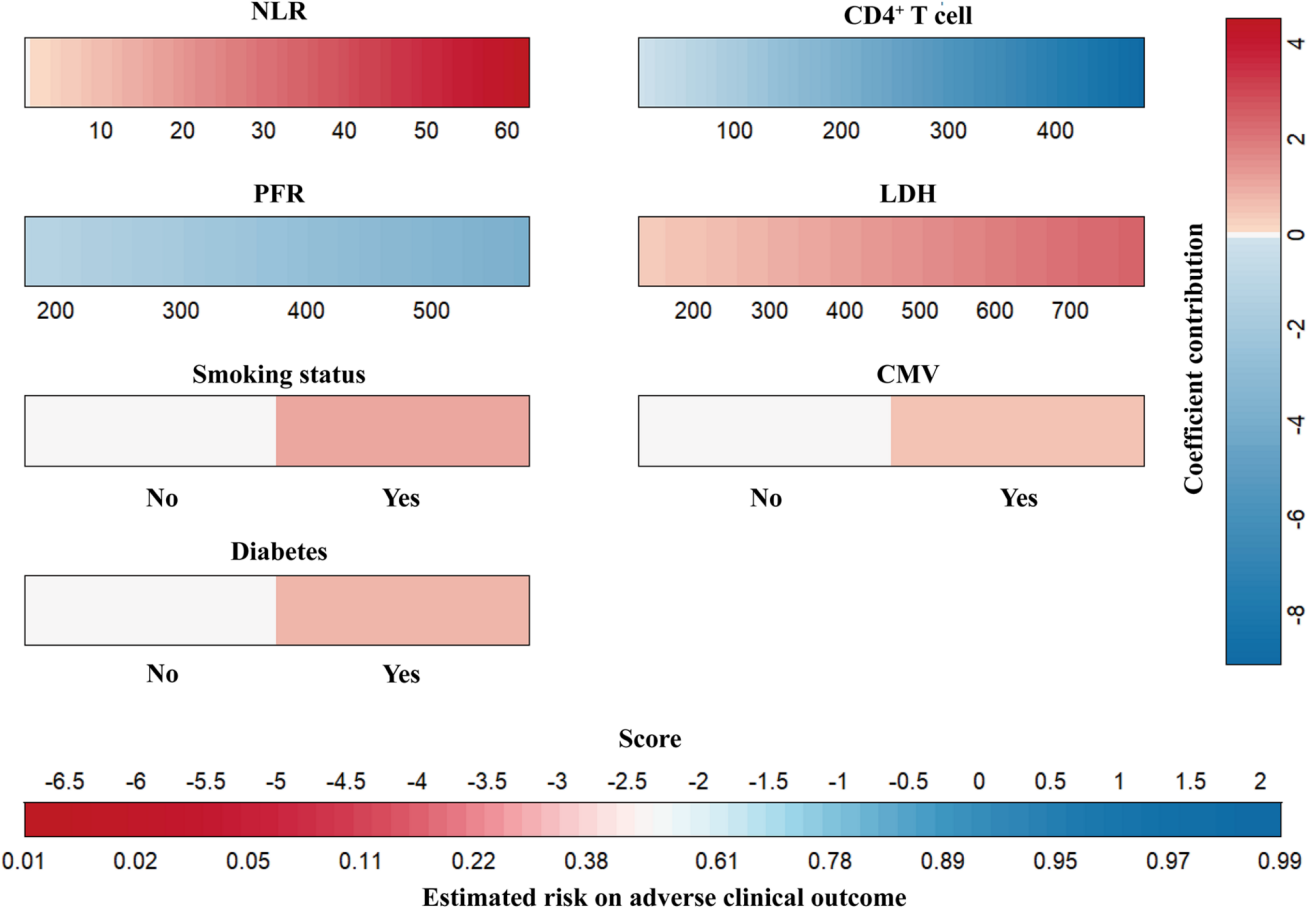
The nomogram for predicting adverse clinical outcomes in non-HIV patients with PJP For each predictor, the variable range is displayed below its corresponding color bar. The color reflects the coefficient contribution to the predictor based on the variable’s value. The contributions are represented as points and can be interpreted using the coefficient contribution scale on the right. The total score, obtained by summing all contributions, is mapped to the estimated risk using the color bar at the bottom. The risk scale transitions from blue (lower risk) to red (higher risk)

**Fig. 4:**
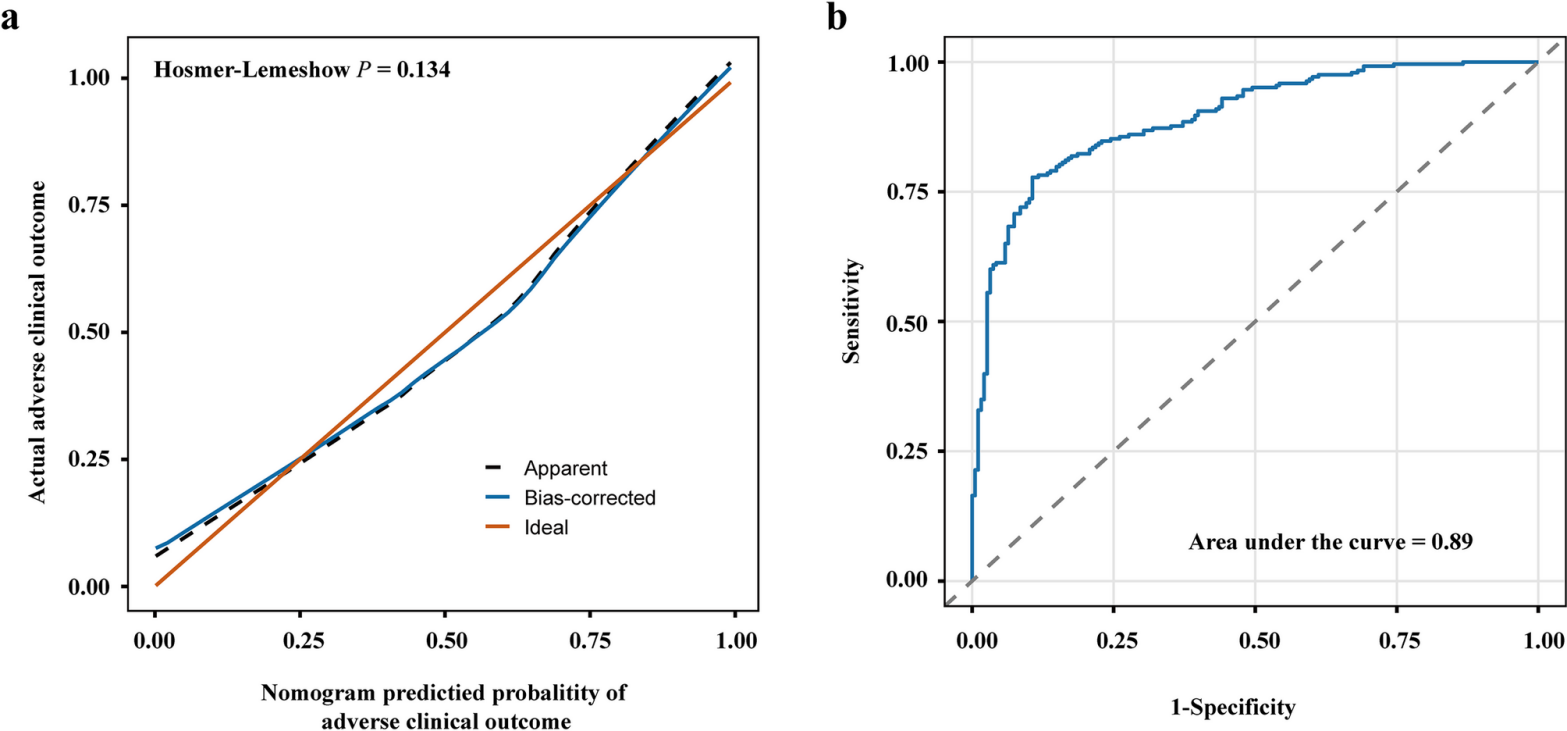
Calibration plot and receiver operating characteristic curve for the predictive model. (**a**) Calibration curves for predictive model. The Hosmer-Lemeshow test *P* = 0.134, suggests a good model fit. (**b**) ROC curve for validating the nomogram. The naive area under curve (AUC): 0.89; optimism-corrected AUC: 0.83

The model incorporated predictors identified through multivariate logistic regression, including smoking status, CMV infection, diabetes, NLR, LDH, PFR, and CD4 ^+^ T cell. A visual nomogram was constructed using regression coefficients to estimate the probability of adverse clinical outcomes based on the total score derived from predictor-specific points (Fig. 3). The model demonstrated a Hosmer-Lemeshow test p-value of 0.134 (Fig. 4a) with a C-index of 0.89 (95%CI: 0.86-0.92) (Fig. 4b). The bias-corrected calibration curve closely aligned with the ideal diagonal, indicating good model calibration. By bootstrapping internal validation, the corresponding internally validated AUC was 0.83 (95%CI: 0.80–0.86).

### Clinical applications of predictive modeling

DCA and CIC were used to assess the model’s clinical utility and reliability. In the DCA plot (Figure S2), the model consistently outperforms the “Treat All” and “Treat None” strategies across a range of threshold probabilities (6-94%). The CIC shows the practical application of the predictive model at varying threshold probabilities (Figure S3). These two curves demonstrate the model’s robust ability to identify high-risk cases accurately. These results further validate the practical applicability of the model in guiding clinical decision-making.

## Discussion

This study developed and validated a predictive nomogram for the early identification of adverse clinical outcomes in non-HIV PJP patients, integrating demographic, key clinical, and laboratory predictors. The nomogram exhibited robust predictive performance, with a C-index of 0.89 and an internally validated AUC of 0.80, suggesting high reliability and accuracy in risk stratification. These findings underscore the importance of integrating diverse patient-specific features to improve risk stratification and prognosis in this heterogeneous population, addressing critical challenges in the management of non-HIV PJP.

The prevalence of immunosuppression among adults has increased significantly, rising from 2.7% in 2013 to 6.6% in 2021, driven by a notable rise in the incidence of immunosuppressive conditions and the expanded use of immunosuppressive therapies [13]. These patients exhibit heightened vulnerability to a wide range of pathogens, including fungal infections. Recent estimates suggest PJP ranks among the top three most prevalent opportunistic fungal infections, alongside invasive candidiasis and invasive aspergillosis [14], with affected patients frequently requiring ICU admission and mechanical ventilation during hospitalization, and experiencing high mortality rates [9]. Our findings are consistent with previous studies [10], further supporting the critical burden of PJP in immunocompromised patients and underscoring the need for timely identification and intervention in this high-risk population.

Currently, the assessment of disease severity in PJP patients largely relies on the Miller criteria, proposed in 1996, which primarily evaluate respiratory features, including respiratory symptoms, oxygenation status, and radiographic findings [15]. However, this scoring system was originally developed for HIV-positive individuals, and is not universally accepted for grading disease severity in non-HIV PJP patients. Several studies have evaluated the performance of pneumonia severity scores such as A-DROP, CURB-65, and Pneumonia Severity Index in this context, but findings suggest limited applicability to non-HIV PJP populations [16]. Other research has examined the prognostic value of ICU-based tools like SOFA and APACHE II scores, yet these remain suboptimal for risk stratification among general ward patients [9, 17]. To address this gap, our study developed and validated a concise, clinically applicable nomogram based on routinely available demographic, clinical, and laboratory variables. This composite scoring approach leverages the synergistic prognostic value of key factors to enable the early identification of high-risk individuals and to inform personalized clinical management strategies in non-HIV PJP.

Among the variables incorporated into our model, we selected the NLR over absolute lymphocyte count as a prognostic marker, given its superior reliability and reproducibility in critically ill patients [18, 19]. The NLR integrates both pro-inflammatory and immunosuppressive components, offering a more comprehensive reflection of immune dysfunction and systemic inflammation in PJP [20]. Our previous study has shown that lymphocyte counts did not differ significantly between survivors and non-survivors in non-HIV PJP, whereas NLR demonstrated stronger prognostic performance, even surpassing CD4^+^ T cell count in predictive accuracy [5]. Additionally, NLR has shown clinical value in various immune-related diseases, including rheumatoid arthritis [21] and psoriasis [22], further supporting its utility as a marker of immune-inflammatory status. We also distinguished between chronic pulmonary diseases–such as chronic obstructive pulmonary disease, asthma, and bronchiectasis–and interstitial lung disease (ILD) in our analysis. Although both categories involve structural lung abnormalities and may impair local respiratory defenses, their immunological characteristics differ substantially. Chronic pulmonary diseases are not inherently immune-mediated and typically do not require immunosuppressive therapy. In contrast, ILD often involves immune-driven inflammation and necessitates long-term glucocorticoid or immunosuppressive treatment, thereby increasing the risk for opportunistic infections like PJP [23].

Drug-induced and iatrogenic factors also play a critical role in shaping immunosuppressive status and increasing susceptibility to PJP [13, 24]. Several guidelines–including those from the German Society for Hematology and Medical Oncology, the American Society of Clinical Oncology, and the Infectious Diseases Society of America–recommend initiating PJP prophylaxis in patients receiving ≥ 20 mg/day of prednisone for ≥ 4 weeks [25, 26]. However, the impact of pre-onset corticosteroid therapy on PJP prognosis has yet to be conclusively determined. Therefore, in this study, we analyzed the association between corticosteroid use within one month prior to admission and adverse hospital outcomes. Although the association did not reach statistical significance, the observed trend toward increased risk suggests potential clinical relevance. Given that long-term corticosteroid therapy is a well-established risk factor for PJP onset and prognosis, it is plausible that cumulative exposure may better capture the risk contribution of corticosteroid therapy. Due to the retrospective nature of our study, we were unable to obtain complete data on corticosteroid dosage and duration, and thus could not calculate cumulative corticosteroid exposure. Notably, a recent multicenter study quantitatively analyzed corticosteroid use and demonstrated a significant association between pre-treatment dosage and 90-day mortality in non-HIV PJP patients, highlighting the prognostic value of corticosteroid burden in this population [27].

In non-HIV populations, PJP often progresses rapidly, and delayed treatment is strongly associated with increased risk of mortality and complications. Our analysis indicated that delayed initiation of TMP-SMX was associated with a higher risk of adverse outcomes. A prospective study has demonstrated that longer time from admission to initiation of PJP therapy is an independent risk factor for in-hospital mortality (OR: 1.11 per additional day, 95% CI: 1.04–1.18) [28]. Several retrospective studies have similarly reported that treatment delays are associated with increased mortality risk [29, 30].Notably, nearly one-quarter of patients in our cohort began anti-Pneumocystis treatment more than 96 h after admission. This highlights a gap between clinical guidelines, which consistently recommend early empirical treatment for high-risk immunocompromised patients and actual clinical practice. However, TMP-SMX often causes side effects, atovaquone is getting more attention as an alternative. Although atovaquone has not yet been approved for clinical use in our country, literature evidence suggests its potential as both a treatment and prophylactic agent in non-HIV immunocompromised patients. Case reports have demonstrated its successful use in patients with renal impairment [31] or prior TMP-SMX-induced toxic epidermal necrolysis [32], and randomized trials have shown favorable safety profiles [33]. Furthermore, recent studies in solid organ transplant, hematologic malignancy, and connective tissue disease populations support the utility of atovaquone as a well-tolerated alternative [34–36]. These findings underscore the need to expand the therapeutic arsenal for PJP, particularly in high-risk patients where first-line therapy is not feasible.

As for adjunctive corticosteroid therapy in non-HIV PJP patients, current evidence remains mixed and inconclusive. One proposed mechanism suggests that in immunocompromised individuals with high fungal burden, antimicrobial-induced lysis of Pneumocystis organisms may release intracellular contents that trigger widespread inflammation. Corticosteroids may attenuate the inflammatory response by limiting further pathogen lysis [37]. However, clinical outcomes have varied. Some studies have reported benefits of adjunctive corticosteroids in non-HIV immunocompromised PJP patients [38], while others—such as a multicenter study in solid organ transplant recipients—found no association between corticosteroid use and mortality [39]. In our analysis, adjunctive corticosteroid use was also associated with a higher risk of adverse outcomes. Collectively, these findings suggest that the role of corticosteroids in non-HIV PJP remains complex and context-dependent. For instance, in patients with immune-mediated inflammatory diseases, the majority are already receiving long-term corticosteroid therapy, potentially limiting the added benefit of high-dose adjunctive regimens [7]. Thus, further investigations are warranted to better delineate which patient subgroups may benefit from corticosteroid co-treatment and under what clinical circumstances.

This study developed and validated a predictive nomogram for identifying adverse clinical outcomes in non-HIV PJP patients, integrating key demographic, clinical, and laboratory predictors such as LDH, NLR, and CD4 ^+^ T cell counts. While the findings provide valuable insights, the study has some limitations. First, the single-center design may limit the generalizability of the results to other populations or healthcare settings; however, it ensured consistent data collection and clinical practices, enhancing internal validity. Second, the retrospective design limited access to precise corticosteroid dose and duration, and although pre-admission use was analyzed qualitatively, cumulative exposure could not be fully evaluated. Third, the use of composite outcomes may provide a comprehensive assessment of clinical deterioration but introduces potential challenges in interpreting the impact of individual endpoints due to overlapping events. Moreover, current therapeutic strategies for non-HIV PJP patients often lead to suboptimal outcomes, emphasizing the urgent need to develop and implement more effective treatment approaches. These considerations collectively highlight the importance of future prospective, multicenter studies not only to validate prognostic tools, but also to rigorously assess and refine therapeutic interventions for this high-risk population.

## Conclusions

Based on regression analysis, our study identified key factors associated with adverse outcomes in non-HIV PJP, including smoking status, CMV infection, diabetes, NLR, LDH, PFR, and lymphocyte subsets. These factors were integrated into a predictive model with high accuracy and reliability, leveraging routine clinical parameters to ensure practicality.

## Electronic supplementary material

Below is the link to the electronic supplementary material.

Supplementary Material 1

Fig. S1: Venn Diagram of IMV, ICU, In-hospital mortality, and 28-day Mortality
Note: IMV, invasive mechanical ventilation; ICU, intensive care unit

Fig. S2: Decision curve analysis curve for predictive model validation
The green line represents the net benefit of the model, the orange line represents the “treat all” strategy, and the solid blue line represents the “treat none” strategy. The bottom color bars indicate the distribution of patients classified as high risk by the nomogram: the blue bar represents patients with adverse outcomes (nomogram relevant), while the orange bar represents those without adverse outcomes (nomogram not relevant)

Fig. S3: Clinical impact curve curves for predictive model validation
The solid blue line represents the total number of patients classified as high risk at each threshold probability, while the dashed red line indicates the number of high-risk patients who experienced the adverse event

## Data Availability

The datasets used and/or analyzed during the current study are available from the corresponding author on reasonable requests.

## Abbreviations

PJP: Pneumocystis *jirovecii* pneumonia
HAART: Highly active antiretroviral therapy
TMP-SMX: Trimethoprim-sulfamethoxazole
ICU: Intensive care unit
LDH: Lactate dehydrogenase
CRP: C-reactive protein
NLR: Neutrophil-to-lymphocyte ratio
IMV: Invasive mechanical ventilation
WBC: White blood cell
PLR: Platelet-to-lymphocyte ratio
BALF: Bronchoalveolar lavage fluid
qPCR: quantitative polymerase chain reaction
IQR: Interquartile range
LASSO: The subsequent least absolute shrinkage and selection operator
AUC: Area Under Curve
DCA: Decision curve analysis
CIC: Clinical impact curve
BUN: Blood urea nitrogen
PFR: PaO_2_/FiO_2_ ratio
CMV: Cytomegalovirus
ILD: Interstitial lung disease
