# Supplementary material for: A nomogram-based clinical prediction model for adverse clinical outcomes in non-HIV Pneumocystis jirovecii pneumonia patients
Source: BMC Pulm Med. 2025 May 17;25:238. doi: 10.1186/s12890-025-03700-2 (PMC12085830; doi:10.1186/s12890-025-03700-2)
Supplement: Supplementary file 1 — Supplementary Material 1 [file 12890_2025_3700_MOESM1_ESM.docx]

**Table S1. The identification and rate of each component of**

**adverse clinical outcome**

| **Patients who had either of the following for condition were considered as adverse clinical outcome:** | **Identification** | **Rate** |
| --- | --- | --- |
| a) received IMV during the hospitalization | IMV records beyond 24 hours after admission | 27.1% (117/431) |
| b) transferred to ICU during the hospitalization | ICU transfer records beyond 24 hours after admission | 45.4% (196/431) |
| c) died during the hospitalization | Death records beyond 24 hours after admission | 26.7% (115/431) |
| d) died for any cause within 28 days after discharge | Death records within 28 days after discharge | 12.5% (54/431) |
| Abbreviations: IMV, invasive mechanical ventilation; ICU, intensive care unit | | |
