# Supplementary figures and images for: A nomogram-based clinical prediction model for adverse clinical outcomes in non-HIV Pneumocystis jirovecii pneumonia patients
Source: BMC Pulm Med. 2025 May 17;25:238. doi: 10.1186/s12890-025-03700-2 (PMC12085830; doi:10.1186/s12890-025-03700-2)

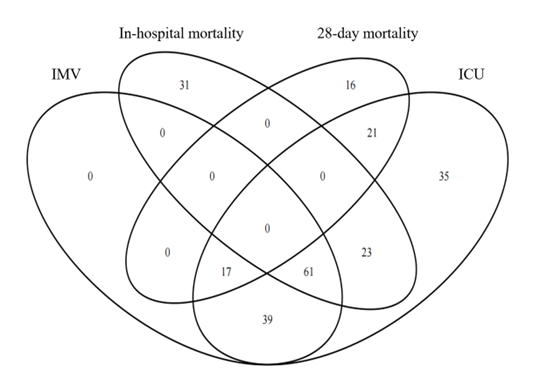

Supplement: Supplementary file 2 — Fig. S1: Venn Diagram of IMV, ICU, In-hospital mortality, and 28-day Mortality Note: IMV, invasive mechanical ventilation; ICU, intensive care unit [file 12890_2025_3700_MOESM2_ESM.png]

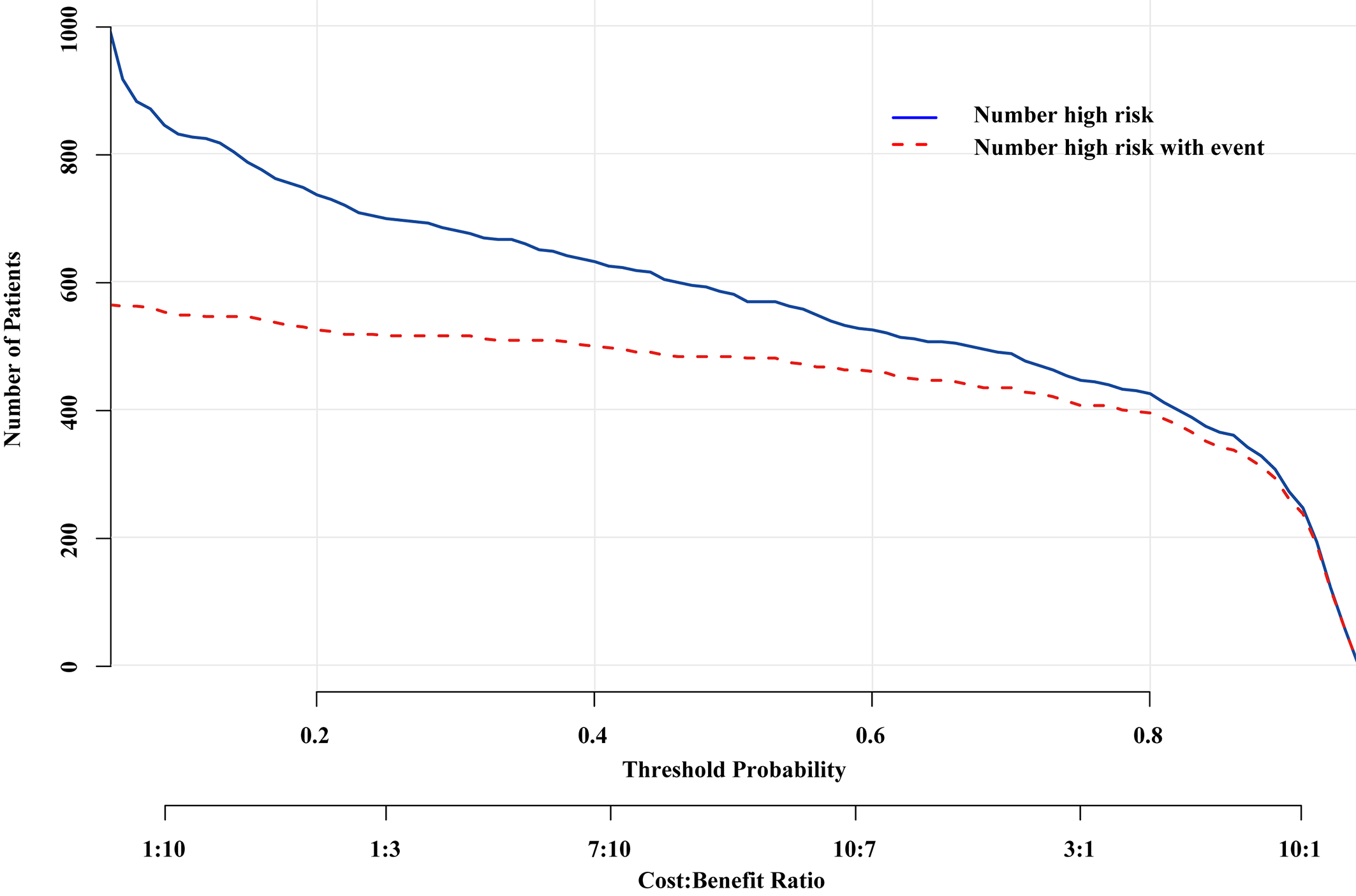

Supplement: Supplementary file 3 — Fig. S2: Decision curve analysis curve for predictive model validation The green line represents the net benefit of the model, the orange line represents the “treat all” strategy, and the solid blue line represents the “treat none” strategy. The bottom color bars indicate the distribution of patients classified as high risk by the nomogram: the blue bar represents patients with adverse outcomes (nomogram relevant), while the orange bar represents those without adverse outcomes (nomogram not relevant) [file 12890_2025_3700_MOESM3_ESM.tif]

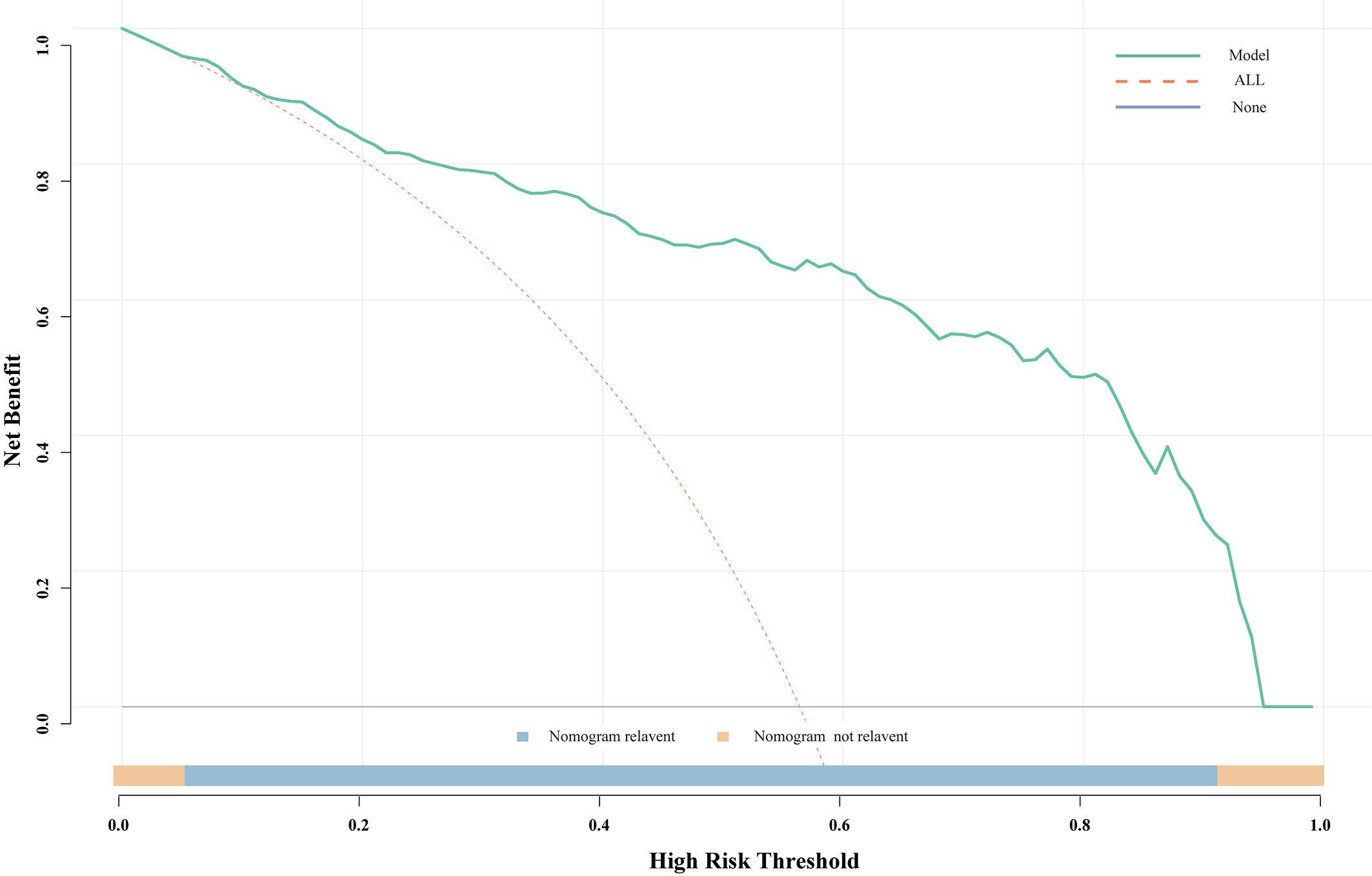

Supplement: Supplementary file 4 — Fig. S3: Clinical impact curve curves for predictive model validation The solid blue line represents the total number of patients classified as high risk at each threshold probability, while the dashed red line indicates the number of high-risk patients who experienced the adverse event [file 12890_2025_3700_MOESM4_ESM.tif]
